# The Chain-Mediating Effect of Obesity, Depressive Symptoms on the Association between Dietary Quality and Cardiovascular Disease Risk

**DOI:** 10.3390/nu15030629

**Published:** 2023-01-26

**Authors:** Shuai Zhang, Limei E, Zhonghai Lu, Yingying Yu, Xuebin Yang, Yao Chen, Xiubo Jiang

**Affiliations:** Department of Epidemiology and Health Statistics, School of Public Health, Qingdao University, Qingdao 266021, China

**Keywords:** obesity, depressive symptoms, dietary quality, HEI-2015, cardiovascular disease, NHANES

## Abstract

In order to explore the relationship between the Healthy Eating Index (HEI-2015) and cardiovascular disease (CVD), and the mediating role of obesity and depressive symptoms, we used the data from the 2011–2018 National Health and Nutrition Examination Survey (NHANES) for further study. A total of 12,644 participants were included in the study. The HEI was derived using NHANES personal food data and USDA Food Pattern Equivalence Database (FPED) dietary data. The risk of cardiovascular disease was determined using the Framingham Heart Study’s multifactorial calculation tool. The weighted multiple logistic regression model was used to explore the association between the HEI-2015 and CVD, and the generalized structural equation was used to explore the mediating effects of obesity and depression, respectively and jointly. Higher HEI-2015 scores were associated with a lower risk of CVD compared to lower quartiles. Obesity, depressive symptoms, and their chain effects all played significant mediating roles in the association between the HEI-2015 and CVD, with proportional mediations of 9.03%, 2.23% and 0.25%, respectively. Our results suggest that higher dietary quality is associated with a lower risk of CVD, mediated by obesity, depressive symptoms, and the chain effect of obesity and depressive symptoms.

## 1. Introduction

Cardiovascular diseases are the leading cause of death globally, with an estimated 17.8 million deaths worldwide and more than 80,000 deaths occurring in the USA, and they account for the highest number of deaths among noncommunicable diseases [1,2]. Atherosclerosis is the most common form of vascular disease and the leading cause of death, accounting for 17.5 million CVD deaths annually (31% of global mortality) [3]. Ameliorating unhealthy eating behaviors has been found to be a new way to reduce the risk of CVD and the health care burden caused by CVD [4,5,6].

Nutritional and dietary factors are closely related to CVD. Previous studies [7,8] have found that Americans have increased their intake of vegetables, fruits, and whole grains over the past few decades and improved the quality of their diets, but not enough overall. It is important to note that because humans do not consume each nutrient group independently, complex food intake may have different synergies [9]. Therefore, in the search for overall dietary quality, it may be better to respect the “dietary pattern” rather than only a single nutrient. Diet quality affects many chronic diseases, including high blood pressure, diabetes, and cardiovascular disease [10]. A recent systematic review [11] found that the HEI may be a good tool for evaluating the effects of multiple diets on cardiometabolic risk interventions. Recent prospective studies found that the Healthy Eating Index−2015 (HEI-2015), which reflects adherence to the 2015–2020 dietary guidelines for Americans, was associated with a reduced risk of CVD and reduced CVD mortality [4,12].

Depression is a widespread and growing global mental health problem [13]. According to the National Institute of Mental Health, in 2017, an estimated 17.3 million adults aged 18 or older in the U.S. had at least one major depressive episode in the past year (6.7% of U.S. adults) [14]. In recent years, the risk of obesity has also become more significant, with the prevalence of being overweight and obese among adults increasing by 28% and 47%, respectively, worldwide between 1980 and 2013 [15]. Two recent prospective studies have shown that high HEI scores are associated with a reduced prevalence of depression [16,17]. A systematic review [18] involving 10 prospective studies and 26 cross-sectional studies found an inverse association between HEI and obesity.

Several prospective studies [19,20,21] have reported that a higher risk of depression is associated with a higher risk of CVD. Meanwhile, several studies [22,23,24] on the association between obesity and CVD risk have shown that obesity is an independent risk factor and increased the mortality of CVD. Therefore, people with a poor diet may become obese or depressed, which in turn can lead to an increased prevalence of CVD.

In conclusion, the above evidence suggests that depression and obesity may be a causal chain between diet and CVD risk. Meanwhile, a review [25] of 25 prospective population-based studies states there is strong evidence that obesity leads to an increase in depression. To date, no complete study has investigated whether obesity, depression, or a combination of the two mediate the association between diet quality and CVD. As a result, our study used the National Health and Nutrition Examination Survey (NHANES) and the Food Patterns Equivalents Database (FPED) diet data to explore the association between diet quality and CVD and further explore whether this association is mediated by obesity and depressive symptoms.

## 2. Materials and Methods

### 2.1. Data Source and Study Sample

The data for the population in this study was from the NHANES (https://www.cdc.gov/nchs/nhanes/index.htm) (accessed on 11 October 2022). The NHANES is a cross-sectional study conducted by the National Center for Health Statistics and the Centers for Disease Control and Prevention that followed a multistage complex sampling design with a two-year cycle. The survey included face-to-face interviews at home (demographic, socioeconomic, dietary, and health-related questions), as well as health examinations (medical and physiological measurements) and laboratory tests (biomarkers of exposure and outcome) at ambulate centers. The data were sampled in a two-year cycle, and all subjects signed the relevant informed consent.

**Figure 1:**
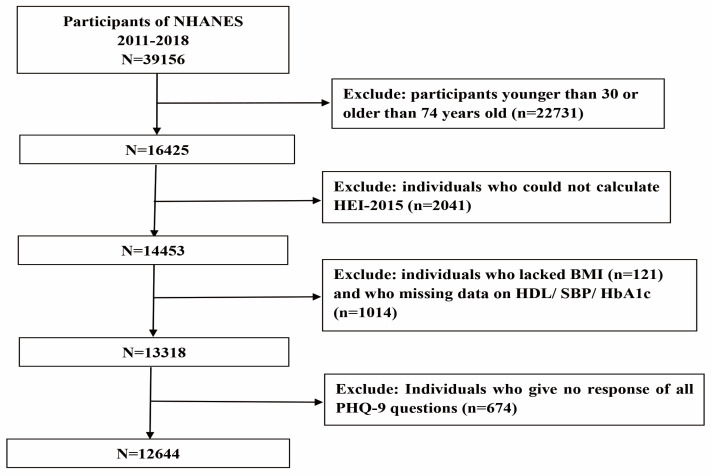
Flow chart of the screening process for the selection of eligible participants.

Data from the NHANES from 2011–2012, 2013–2014, 2015–2016, and 2017–2018 were selected for this study, which included a total of 39,156 participants. A total of 22,731 participants younger than 30 or older than 74 years of age and 2041 participants unable to calculate their HEI-2015 were excluded. A total of 121 individuals were eliminated because of missing BMI data, and 1014 participants were missing the data to calculate the risk of CVD. An additional 674 individuals who did not have a complete Patient Health Questionnaire PHQ-9 response were excluded. In the end, 12,644 individuals were included in the study (Figure 1).

### 2.2. Dietary Quality

The Healthy Eating Index (HEI) is used to assess the consistency of any group of foods with key dietary quality recommendations made by the Dietary Guidelines for Americans (DGA). The latest version of the HEI is the HEI-2015. The HEI-2015 consists of 13 components [26]. Nine of those recommended components (higher intake means a higher score) include total fruits, whole fruits, total vegetables, greens and beans, whole grains, dairy, total protein foods, seafood and plant proteins, and fatty acids. Four moderation components (lower intake means a higher score) include refined grains, sodium, added sugars, and saturated fats. The maximum score is 100. The higher the scores of the HEI-2015, the higher diet quality of participants.

The NHANES individual food data and Food Patterns Equivalents Database (FPED) diet data were used to estimate the intake of the thirteen food components used to construct the HEI-2015. Each food is classified according to the USDA Codex Alimentarius. The total score of the HEI-2015 was calculated by SAS code.

### 2.3. Depressive Symptoms

The outcome variable was depressive symptoms. The Patient Health Questionnaire (PHQ-9) is a nine-item scale. Each of the nine questions consists of four answers: “none at all“, “a few days”, “more than half of the days”, and “almost every day”, with a score range of 0 to 3 for each question. The total score is a composite of the nine question scores with a maximum score of 27 points and a cut-off of 10 points [27]. According to the cut-off value, participants were divided based on those with or without depressive symptoms.

### 2.4. Obesity

According to World Health Organization standards, general obesity is defined as a BMI [BMI = weight (kg)/height (m)^2^] ≥ 30 kg/m^2^.

### 2.5. Cardiovascular Disease

The Framingham Heart Study, a tool that synthesizes vascular risk factor information to produce estimates of an individual patient’s absolute cardiovascular disease risk, was used to predict the end point (also known as global cardiovascular disease risk) [28]. CVD risk is a composite outcome that includes coronary heart disease (including cardiac death, myocardial infarction, coronary insufficiency, and angina pectoris), cerebrovascular events (such as ischemic stroke, hemorrhagic stroke, and transient ischemic attack), and peripheral artery disease. The sex-specific Framingham general CVD risk score is defined by six different measures, which are age, total cholesterol, HDL cholesterol, systolic blood pressure, whether they are being treated for high blood pressure, and diabetes and smoking status. The tool is recommended for the age group 30–74, and the risk of heart disease is divided into two categories by predicting a 10-year CVD risk score: low (≤20%) and high (>20%) [28].

### 2.6. Covariates

Trained NHANES investigators obtained demographic information from participants living in sample areas. In order to control the effect of potential confounders, we included the following covariates: age (actual value), sex (man or woman), ratio of family income to poverty (actual value), smoking (never smoker: lifetime intake of no more than 100 cigarettes; former smoker: lifetime intake of more than 100 cigarettes but current serum cotinine does not reach the threshold; current smoker: lifetime intake of more than 100 cigarettes and current serum cotinine reaches the threshold), drink (yes or no), hypertension (yes or no), work activity (vigorous activity, moderate activity, or low activity), recreational activity (vigorous activity, moderate activity, or low activity), marital status (married/living with partner or widowed/divorced/separated/never married), education of household referent (less than high school, high school, or more than high school), race/ethnicity (Mexican American, Other Hispanic, Non-Hispanic White, Non-Hispanic Black, and Other Race), diabetes (yes: self-reported physician diagnosis or glycosylated hemoglobin level (HbA1c) ≥ 6.5% or fasting blood glucose (FBG) ≥ 7.0 mmol/L or taking hypoglycemic drugs to lower blood sugar; or no), cycle of the participants (2011–2012, 2013–2014, 2015–2016, or 2017–2018).

The threshold for serum cotinine, which is used to distinguish former smokers and current smokers, were for non-Hispanic white > 4.85 ng/mL, non-Hispanic Black > 5.92 ng/mL, Mexican American > 0.84 ng/mL, and other > 3.08 ng/mL [29]. Whether or not to receive treatment for hypertension was based on a self-administered questionnaire. The serum HDL-C and serum TC were measured using the Roche/Hitachi Modular P Chemistry Analyzer, with HDL-C measured using a magnesium/dextran sulfate method and serum TC measured using a completely enzymatic method [30,31].

### 2.7. Sensitivity Analysis

To make our results more representative, we performed a sensitivity analysis. We considered that the use of prescription drugs might have an effect on the mediated outcomes, and therefore, the use of the depression treatment scale was obtained through the prescription drug scale. Depression prescription drug use was obtained through personal interviews with prescription drug use data. We screened participants who used “major depressive disorder, single episode” and “major depressive disorder, recurrent”. We performed a sensitivity analysis on patients who took prescription drugs but were identified as having no depressive symptoms by the PHQ-9 scale and those who were previously identified as having depressive symptoms.

### 2.8. Statistical Analysis

Stata 12.0 (Stata Corporation, College Station, TX, USA) and SAS version 9.4 (SAS Institute, Inc., Cary, NC, USA) were used for the entire statistical analysis. All analyses were adjusted for survey design and weighted variables to account for the complex sampling design. This study combined four cycles of NHANES; considering the complex sampling design, we established a new weight (the original 2-year sample weight divided by 4) according to the guidelines of NHANES.

The basic characteristics of classified variables were described by percentage, and the basic characteristics of continuous variables were described by mean and standard deviation. To analyze differences between continuous data, the Kruskal–Wallis test or one-way analysis of variance (ANOVA) were used, while the chi-square test was used to analyze differences between classified data. Diet quality was categorized based on quartiles (Q1: ≤ 25th percentile, Q2: > 25 to 50th percentile, Q3: > 50 to 75th percentile, Q4: > 75th percentile). Weighted multivariate logistic regression was used to explore the relationship between the HEI-2015 and CVD. Model 1 was adjusted for age and sex. Model 2 had additional adjustments for race/ethnicity, education, marital status, poverty ratio, drinking status, work activities, and recreational activities. The generalized structural equation (GSEM) and bootstrap method were used to explore the mediating effect of obesity and depressive symptoms on the association between the HEI-2015 and CVD. A two-sided *p* < 0.05 was considered statistically significant.

## 3. Results

Table 1 shows baseline characteristics of participants in terms of CVD. A total of 18,228 participants were included in our study. There were significant differences between people with CVD and people without CVD in the distribution of age, gender, race/ethnicity, degree of education, ratio of family income to poverty, marital status, smoking status, hypertension, HDL cholesterol, work physical activity, recreational physical activity, obesity, and diabetes status. There was no significant difference between age group and total cholesterol.

Table 2 shows the associations among the HEI-2015, obesity, and depressive symptoms with CVD. In exploring the association between the HEI-2015 and CVD, we found that compared with the control group Q1, the associations between the Q2, Q3, and Q4 groups and CVD were statistically significant (OR_Q2_ = 0.774, 95% CI: 0.630, 0.950; OR_Q3_ = 0.591, 95% CI: 0.462, 0.755; OR_Q4_ = 0.456, 95% CI: 0.363, 0.572) after being adjusted for age and sex. After further adjusting for race/ethnicity, education, marital status, poverty ratio, drinking status, work activities, and recreational activities (Model 2), the adjusted ORs with 95% CIs of the HEI-2015 Q3 and Q4 groups for the risk of CVD were still significant (OR_Q3_ = 0.690, 95% CI: 0.539, 0.882; OR_Q4_ = 0.632, 95% CI: 0.501, 0.797). When adjusted for age and sex, the adjusted ORs with 95% CIs of depressive symptoms and obesity for the risk of CVD were significant (OR_depressive symptoms_ = 1.925, 95% CI: 1.505, 2.642; OR_obesity_ = 2.035, 95% CI: 1.766, 2.345). After the full adjustment in Model 2, we found that depressive symptoms and obesity were associated with CVD (OR_depressive symptoms_ = 1.369, 95% CI: 1.039, 1.803; OR_obesity_ = 1.914, 95% CI: 1.632, 2.244).

Table 3 shows the associations between the HEI-2015 and depressive symptoms and obesity, respectively. After adjustments for age and sex, the adjusted ORs with 95% CIs of the HEI-2015 for depressive symptoms were significant (OR_Q2_ = 0.780, 95% CI: 0.629, 0.968; OR_Q3_ = 0.521, 95% CI: 0.399, 0.680; OR_Q4_ = 0.361, 95% CI: 0.276, 0.473). After adjusting for all confounding variables, we found that compared with the Q1 group, Q3 and Q4 in the HEI-2015 were statistically significant in association with depressive symptoms (OR_Q3_ = 0.682, 95% CI: 0.512, 0.908; OR_Q4_ = 0.553, 95% CI: 0.417, 0.735). Compared with the control group, the associations between the Q2, Q3, and Q4 groups of the HEI-2015 and obesity were statistically significant (OR_Q2_ = 0.806, 95% CI: 0.691, 0.941; ORQ3 = 0.676, 95% CI: 0.591, 0.772; ORQ4 = 0.519, 95% CI: 0.442, 0.610).

Table 4 shows the correlation between the scores of 13 components of the HEI-2015 and the risk of CVD. After adjusting for all the variables, we found that the increased intake of greens and beans, total fruits, fatty acids, and seafood and plant proteins was associated with low risk of CVD (OR_greens and beans_ = 0.963, 95% CI: 0.929, 0.999; OR_seafood and plant proteins_ = 0.956, 95% CI: 0.923, 0.989; OR_fatty acid_ = 0.964, 95% CI: 0.942, 0.986). The decreased intake of sodium, refined grains, and saturated fats was associated with low risk of CVD (OR_sodium_ = 0.964, 95% CI: 0.942, 0.986; OR_refined grains_ = 0.974, 95% CI: 0.952, 0.997; OR_saturated fats_ = 0.972, 95% CI: 0.951, 0.994). No significant correlations were found between total vegetables, total fruits, whole fruits, whole grains, dairy, total protein foods, and added sugars and the risk of CVD.

**Figure 2:**
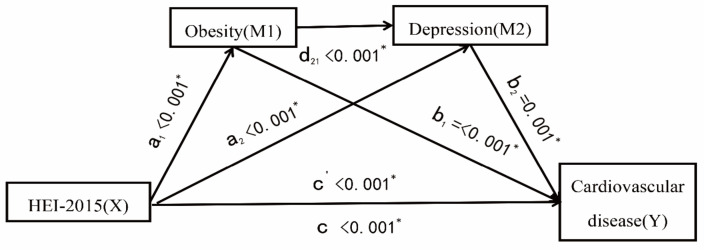
Association of HEI-2015 (X) and cardiovascular disease (Y) mediated by obesity (M1) and depressive symptoms (M2). a_1_ represents the effect of M1 on X; a_2_ represents the effect of M2 on X; b_1_ represents the effect of Y on M1; b_2_ represents the effect of Y on M2; d_21_ represents the effect of M2 on M1; C represents the direct effect, and c’ represents the total effect. * Solid lines indicate statistically significant associations; dashed lines indicate no statistically significant associations. Adjusted for age and sex, race/ethnicity, education, marital status, poverty ratio, drinking status, work activities, and recreational activities.

Based on the previous investigation, we investigated the relationship between the HEI-2015 and CVD mediated by obesity and depressive symptoms, separately and jointly, and the results are shown in Figure 2 and Table 5. We discovered that after controlling for confounding factors, the overall effect of the association between the HEI-2015 and CKD was statistically significant (*p* < 0.05). We found that obesity and depressive symptoms as mediators independently mediated the association between the HEI-2015 and CVD with statistical significance (*p* < 0.05), implying that obesity and depressive symptoms independently mediate the association between the HEI-2015 and CVD (β_obesity_ =−0.00013, 95% CI: −0.00020, −0.00006; β_depressive symptoms_ = −0.00003, 95% CI: −0.00005, −0.00001), and the mediating proportion was 9.03% and 2.23%, respectively. We further explored the association between the HEI-2015 and CVD co-mediated by obesity and depressive symptoms, and we found that the mediating effect was statistically significant (β_joint_ = −0.000003, 95% CI: −0.000006, −0.0000009).

Table 6 shows the results of depressive symptoms, obesity, and their chain mediation effect on the association between the HEI-2015 and the risk of CVD after adjusting for depressive symptoms through prescription drug use data. The results are still significant whether there were two simple mediations or one chain mediation (*p* < 0.05).

## 4. Discussion

This cross-sectional study used NHANES data from 2011–2012, 2013–2014, 2015–2016, and 2017–2018 cycles to explore the relationship between the HEI-2015 and the risk of CVD and the mediating role of obesity and depressive symptoms. We found that higher diet quality was associated with a lower risk of CVD. In addition, obesity, depressive symptoms, and the combined effects of obesity and depressive symptoms all play a partial mediating role in the relationship between the HEI-2015 and CVD. After the sensitivity analysis, we found that our conclusions were still solid.

Our findings on the relationship between the HEI-2015 and the risk of CVD are consistent with previous studies. A prospective study [4] involving 12,413 participants found that adherence to the HEI-2015 and other dietary patterns may reduce the risk of cardiovascular disease, cardiovascular mortality, and all-cause mortality. Other recent prospective studies [32,33] have produced consistent results with what we have found. The Dietary Patterns Methods Project used a standardized approach to synthesize findings from three cohorts (NIH-American Association of Retired Persons Diet and Health Study, the Multiethnic Cohort, and the Women’s Health Initiative Observational Study) to evaluate the association of various diet quality scores with the risk of CVD death and found an 11–28% reduction in the risk of death from all causes, CVD, and cancer [34]. Existing evidence suggests that a complex set of several nutrients may interact with genetic factors to influence CVD risk, making it particularly important to focus on whole foods and dietary patterns [35].

The influence of dietary quality on CVD may be mediated in part by obesity and depressive symptoms. We found a significant negative association between the HEI-2015 score and depressive symptoms, and an increase in depressive symptoms was associated with CVD risk. After analyzing the correlation between the 13 components of the HEI-2015 and CVD, we found that the increased intake of greens and beans, fatty acids, and seafood and plant proteins and the decreased intake of sodium, refined grains, and saturated fats are associated with low CVD risk. A prospective study of 521,120 U.S. retirees followed for 16 years found that saturated fatty acid intake was associated with the risk of CVD [36]. A meta-analysis of randomized controlled studies demonstrated a causal relationship between diet quality and depressive symptoms [37]. Previous studies have shown that higher dietary sodium intake increases the risk of depression risk factors, such as high blood pressure, which in turn may affect neurological function [38,39].

We found that the HEI-2015 score was negatively associated with obesity, and an increase in obesity level was associated with CVD risk. Previous results in animal studies have found that high levels of sodium, saturated fat, and added sugars negatively affect brain function by damaging the frontal, limbic, and hippocampal regions of the brain [40], and that high intakes of these substances are strongly linked to obesity [41,42,43]. Greens and beans are rich in tryptophan, which is involved in the synthesis of the neurotransmitter serotonin, and adequate serotonin has been linked to preventing brain damage and reducing the risk of obesity [44,45,46]. Beans are also a good source of plant protein. Fruits contain polyphenols and nutrients with anti-inflammatory properties, which may be linked to lower levels of depression and obesity [47,48]. Compared to people of normal weight, people with obesity are at greater risk of developing depressive symptoms [49].

Obesity and depressive symptoms are both important risk factors for CVD. Depressive symptoms may be risk factors for an increased risk of CVD [20,50]. An exploration of early-onset cardiovascular disease mortality in the United States and Australia that linked death certificate data to the prevalence of obesity in a cohort found that being overweight or obese may adversely affect CVD mortality trends [51]. Depressive symptoms are risk factors for cardiovascular disease, and comorbidities of depressive symptoms with existing CVD worsen the prognosis of patients with CVD [52]. These studies are consistent with our exploration. Previous prospective studies have shown that depressive symptoms interact with CVD risk factors (current smoking, hypertension, and BMI) in a statistically significant way, suggesting that depressive symptoms may increase the association between CVD risk factors and CVD outcomes [53]. Obesity is a risk factor for CVD and is closely related to other risk factors of CVD. Reducing obesity should be an important component of a CVD management strategy [54].

Taking all the above evidence together, we found that diet quality may increase the risk of cardiovascular disease by influencing obesity and depression symptoms. Although diet quality is significantly associated with obesity, depressive symptoms, and the risk of CVD, studies linking the four groups are fairly rare. To our knowledge, this is the first study to fully investigate the mediating role of obesity and depressive symptoms in the relationship between diet quality and the risk of CVD. Therefore, active dietary interventions may help reduce the occurrence of obesity and depressive symptoms and, thus, re-duce the risk of cardiovascular disease.

Our research has some advantages. To our knowledge, this is the first complete exploration of the mediating effects of obesity and depression separately and together in the association between diet quality and cardiovascular disease. Second, we included a large number of people in our study, and the NHANES used complex stratified sampling, making the results nationally representative. At the same time, the shortcomings in our study should not be ignored. First, because this is a cross-sectional study, we cannot draw conclusions about cause and effect. Second, although we control most of the covariates as much as possible, there are still some unknown or difficult to measure data that we cannot control.

## 5. Conclusions

Our findings suggest that better diet quality may reduce the level of obesity and depressive symptoms and, thus, the risk of cardiovascular disease. At the same time, we found that dietary interventions may be an economically viable means to control obesity, depression, and, therefore, cardiovascular disease.

## Figures and Tables

**Table 1:** Basic characteristics of the included sample (*n* = 12,644).

Characteristics	All Participants	Quartile of HEI-2015				
		Q1(0–44.12)	Q2(44.12–53.58)	Q3(53.58–63.50)	Q4(63.50–100)	*p* Value
No. of participants	12,644	3161	3161	3161	3161	
Age (mean±SD) ^a^	51.46 ± 12.61	49.25 ± 12.56	50.99 ± 12.51	51.72 ± 12.52	53.86 ± 12.44	0.961
Gender (%) ^b^						<0.001
Men	6219(49.2)	1752(55.6)	1597(50.5)	1505(47.6)	1365(43.2)	
Women	6425(50.8)	1409(44.6)	1564(49.5)	1656(52.4)	1796(56.8)	
Race/ethnicity (%) ^b^						<0.001
Mexican American	1813(14.3)	426(13.5)	478(15.1)	494(15.6)	415(13.1)	
Other Hispanic	1405(11.1)	273(8.6)	311(9.8)	371(11.7)	450(14.2)	
Non-Hispanic White	4683(37.0)	1372(43.4)	1160(36.7)	1115(35.3)	1036(32.8)	
Non-Hispanic Black	2858(22.6)	793(25.1)	793(25.1)	685(21.7)	587(18.6)	
Other Race	1885(14.9)	297(9.4)	419(13.3)	496(15.7)	673(21.3)	
Degree of education (%) ^b^						<0.001
Less than high school	2668(21.1)	768(24.3)	706(22.3)	664(21.0)	530(16.8)	
High school	2772(21.9)	848(26.8)	772(24.4)	653(20.7)	499(15.8)	
More than high school	7200(57.0)	1544(48.9)	1682(53.2)	1844(58.3)	2130(67.4)	
Ratio of family income to poverty (PIR) (mean ± SD) ^c^	2.62 ± 1.66	2.22 ± 1.53	2.48 ± 1.63	2.69 ± 1.66	3.08 ± 1.69	<0.001
Marital status (%) ^b^						<0.001
Married	8176(64.7)	1907(60.4)	1995(63.2)	2096(66.3)	2178(68.9)	
Other	4462(35.3)	1252(39.6)	1163(36.8)	1065(33.7)	983(31.1)	
Smoke (%) ^b^						<0.001
Nonsmokers	6915(54.7)	1419(44.9)	1629(51.6)	1800(56.9)	2067(65.5)	
Former smoker	2740(21.7)	554(17.5)	684(21.7)	740(23.4)	762(24.1)	
Current smoker	2979(23.6)	1185(37.5)	844(26.7)	621(19.6)	329(10.4)	
Work physical activity (%) ^b^						<0.001
Vigorous activity	2733(21.6)	884(28.0)	736(23.3)	598(18.9)	515(16.3)	
Moderate activity	2644(20.9)	659(20.8)	672(21.3)	675(21.4)	638(20.2)	
Low activity	7267(57.5)	1618(51.2)	1753(55.5)	1888(59.7)	2008(63.5)	
Recreational physical activity (%) ^b^						<0.001
Vigorous activity	2723(21.5)	486(15.4)	584(18.5)	715(22.6)	938(29.7)	
Moderate activity	3416(27.0)	716(22.7)	782(24.7)	870(27.5)	1048(33.2)	
Low activity	6505(51.4)	1959(62.0)	1795(56.8)	1576(49.9)	1175(37.2)	
Obesity (%) ^b^						<0.001
No	7219(57.1)	1578(49.9)	1746(55.2)	1819(57.5)	2076(65.7)	
Yes	5425(42.9)	1583(50.1)	1415(44.8)	1342(42.5)	1085(34.3)	
Diabetes (%) ^b^						<0.001
No	9971(78.9)	2484(78.6)	2507(79.3)	2457(77.7)	2523(79.8)	
Yes	2673(21.1)	677(21.4)	654(20.7)	704(22.3)	638(20.2)	
Depressive symptoms (%) ^b^						<0.001
No	11447(90.5)	2757(87.2)	2836(89.7)	2883(91.2)	2971(94.0)	
Yes	1197(9.5)	404(12.8)	325(10.3)	278(8.8)	190(6.0)	
Cardiovascular risk (%) ^b^						<0.001
Low	9727(76.9)	2382(75.4)	2409(76.2)	2445(77.3)	2491(78.8)	
High	2917(23.1)	779(24.6)	752(23.8)	716(22.7)	670(21.2)	
Total cholesterol (%) ^b^						0.087
No	7210(57.0)	1864(59.0)	1776(56.2)	1786(56.5)	1784(56.4)	
Yes	5434(43.0)	1297(41.0)	1385(43.8)	1375(43.5)	1377(43.6)	
HDL cholesterol (%) ^b^						<0.001
No	10166(80.4)	2345(74.2)	2542(80.4)	2585(81.8)	2694(85.2)	
Yes	2478(19.6)	816(25.8)	619(19.6)	576(18.2)	467(14.8)	
Hypertension (%) ^b^						<0.001
No	5135(40.6)	1239(39.2)	1221(38.6)	1301(41.2)	1374(76.9)	
Yes	7509(59.4)	1922(60.8)	1940(61.4)	1860(58.8)	1787(56.5)	

^a^ *p* value was tested by the Kruskal–Wallis test; ^b^ *p* value was tested by chi-square test; ^c^ *p* value was tested by one-way analysis of variance (ANOVA).

**Table 2:** Weighted odds ratios (ORs) with 95 percent confidence intervals (CIs) for the HEI-2015, obesity, depressive symptoms, and the risk of CVD.

	Crude ^a^			Model 1 ^a^			Model 2 ^a^		
	t	*p* Value	OR ^b^ (95% CI)	t	*p* Value	OR (95% CI)	t	*p* Value	OR (95% CI)
HEI-2015									
Q1(0–44.12)	Ref.			Ref.			Ref.		
Q2(44.12–53.58)	−0.46	0.648	0.963(0.815, 1.137)	−2.49	0.015	0.774(0.630, 0.950)	−1.97	0.054	0.810(0.653, 1.003)
Q3(53.58–63.50)	−2.83	0.006	0.769(0.639, 0.926)	−4.29	<0.001	0.591(0.462, 0.755)	−3.01	0.004	0.690(0.539, 0.882)
Q4(63.50–100)	−2.71	0.009	0.783(0.654, 0.938)	−6.92	<0.001	0.456(0.363, 0.572)	−3.96	<0.001	0.632(0.501, 0.797)
Depressive symptoms	2.05	0.044	1.230(1.005, 1.506)	5.32	<0.001	1.925(1.505, 2.642)	2.28	0.026	1.369(1.039, 1.803)
Obesity	6.94	<0.001	1.590(1.391, 1.816)	10.02	<0.001	2.035(1.766, 2.345)	8.16	<0.001	1.914(1.632, 2.244)

^a^ Calculated using logistic regression. Model 1 adjusted for age and sex. Model 2 adjusted for age and sex, race/ethnicity, education, marital status, poverty ratio, drinking status, work activities, recreational activities, and cycle of the participants. ^b^ OR: odds ratio.

**Table 3:** Weighted odds ratios (ORs) with 95 percent confidence intervals (CIs) for the HEI-2015 and depressive symptoms and obesity, respectively.

	Crude ^a^			Model 1 ^a^			Model 2 ^a^		
	t	*p* Value	OR ^b^ (95%CI)	t	*p* Value	OR (95%CI)	t	*p* Value	OR (95%CI)
**Depressive symptoms** HEI−2015									
Q1(0–44.12)	Ref.			Ref.			Ref.		
Q2(44.12–53.58)	−2.07	0.043	0.802(0.648, 0.993)	−2.30	0.025	0.780(0.629, 0.968)	−1.08	0.285	0.884(0.703, 1.111)
Q3(53.58–63.50)	−4.55	<0.001	0.555(0.429, 0.719)	−4.89	<0.001	0.521(0.399, 0.680)	−2.67	0.009	0.682(0.512, 0.908)
Q4(63.50–100)	−7.25	<0.001	0.392(0.303, 0.508)	−7.53	<0.001	0.361(0.276, 0.473)	−4.18	<0.001	0.553(0.417, 0.735)
**Obesity** HEI−2015									
Q1(0–44.12)	Ref.			Ref.			Ref.		
Q2(44.12–53.58)	−3.11	0.003	0.788(0.676, 0.918)	−3.32	0.002	0.774(0.663, 0.903)	−2.79	0.007	0.806(0.691, 0.941)
Q3(53.58–63.50)	−7.40	<0.001	0.618(0.542, 0.704)	−7.51	<0.001	0.600(0.523, 0.687)	−5.87	<0.001	0.676(0.591, 0.772)
Q4(63.50–100)	−12.44	<0.001	0.432(0.377, 0.494)	−12.62	<0.001	0.413(0.359, 0.475)	−8.12	<0.001	0.519(0.442, 0.610)

^a^ Calculated using logistic regression. Model 1 adjusted for age and sex. Model 2 adjusted for age and sex, race/ethnicity, education, marital status, poverty ratio, drinking status, work activities, recreational activities, and cycle of the participants. ^b^ OR: odds ratio.

**Table 4:** Weighted odds ratios (ORs) with 95 percent confidence intervals (CIs) for diet components and the risk of CVD.

	Cardiovascular Disease ^a^					
	Crude Model ^b^		Model 1 ^c^		Model 2 ^d^	
	OR(95% CI)	*p* Value	OR(95% CI)	*p* Value	OR(95% CI)	*p* Value
Total vegetables	0.952(0.908, 0.998)	0.043	0.903(0.854, 0.954)	<0.001	0.975(0.916, 1.037)	0.412
Greens and beans	0.938(0.912, 0.965)	<0.001	0.933(0.900, 0.966)	<0.001	0.963(0.929, 0.999)	0.042
Total fruits	0.981(0.950, 1.014)	0.245	0.923(0.887, 0.962)	<0.001	0.959(0.918, 1.001)	0.058
Whole fruits	0.988(0.959, 1.018)	0.426	0.924(0.888, 0.961)	<0.001	0.964(0.921, 1.009)	0.116
Whole grains	1.015(0.998, 1.032)	0.087	0.971(0.949, 0.994)	0.014	0.991(0.968, 1.014)	0.416
Dairy	0.964(0.942, 0.988)	0.003	0.981(0.951, 1.012)	<0.216	1.006(0.975, 1.039)	0.691
Total protein foods	1.075(1.009, 1.146)	0.025	0.970(0.896, 1.051)	0.452	0.997(0.917, 1.084)	0.950
Seafood and plant proteins	0.957(0.931, 0.985)	0.003	0.909(0.878, 0.941)	<0.001	0.956(0.923, 0.989)	0.011
Fatty acid	0.975(0.958, 0.992)	0.005	0.960(0.937, 0.984)	0.001	0.964(0.942, 0.986)	0.002
Sodium ^e^	0.978(0.959, 0.997)	0.022	0.967(0.946, 0.988)	0.003	0.962(0.939, 0.986)	0.003
Refined grains ^e^	0.998(0.983, 1.014)	0.845	0.958(0.939, 0.977)	<0.001	0.974(0.952, 0.997)	0.026
Saturated fats ^e^	0.969(0.953, 0.986)	<0.001	0.978(0.954, 1.002)	0.073	0.972(0.951, 0.994)	0.014
Added sugars ^e^	1.016(0.991, 1.040)	0.208	0.961(0.933, 0.990)	0.010	0.990(0.957, 1.023)	0.521

^a^ Calculated using logistic regression. ^b^ Crude model included only diet quality and did not adjust for covariates. ^c^ Model 1 adjusted for age and sex. ^d^ Model 2 adjusted for age and sex, race/ethnicity, education, marital status, poverty ratio, drinking status, work activities, recreational activities, and cycle of participants. ^e^ Moderate ingredients, a lower intake means a higher score.

**Table 5:** The mediating proportion of obesity and depressive symptoms on the association between the HEI-2015 and the risk of cardiovascular disease.

Model Pathways	Mediating Effect	
	β (95%CI)	Proportion Mediated (%)
Total effect	−0.0014(−0.0019, −0.0009) ***	100
Direct effect	−0.0012(−0.0017, −0.0008) ***	88.74
Indirect effect via obesity	−0.00013(−0.00020, −0.00006) ***	9.03
Indirect effect via depressive symptoms	−0.00003(−0.00005, −0.00001) *	2.23
Indirect effect via obesity and depressive symptoms	−0.000003(−0.000006, −0.0000009) **	0.25

Adjusted for age and sex, race/ethnicity, education, marital status, poverty ratio, drinking status, work activities, recreational activities, and cycle of participants. * *p* < 0.05, ** *p* < 0.01, *** *p* < 0.001.

**Table 6:** After adjusting for depressive symptoms through prescription drug use data, the mediating proportion of obesity and depressive symptoms on the association between the HEI-2015 and the risk of cardiovascular disease.

Model Pathways	Mediating Effect	
	β (95%CI)	Proportion Mediated (%)
Total effect	−0.0014(−0.0018, −0.0010) ***	100
Direct effect	−0.0013(−0.0016, −0.0009) ***	89.64
Indirect effect via obesity	−0.00012(−0.00016, −0.00007) ***	8.40
Indirect effect via depressive symptoms	−0.00003(−0.00005, −0.00001) *	1.96
Indirect effect via obesity and depressive symptoms	−0.000004(−0.000008, −0.0000008) **	0.30

Adjusted for age and sex, race/ethnicity, education, marital status, poverty ratio, drinking status, work activities, recreational activities, and cycle of participants. * *p* < 0.05, ** *p* < 0.01, *** *p* < 0.001.

## Data Availability

The data are available at https://www.cdc.gov/nchs/nhanes/index.htm (accessed on 22 October 2022).
